# 基于Co-BDC-NH_2_的二维手性金属有机框架纳米片用作气相色谱固定相

**DOI:** 10.3724/SP.J.1123.2024.06004

**Published:** 2025-04-08

**Authors:** Meifang YANG, Kangni ZHENG, Yixing LONG, Yijie LI, Xueping WANG, Junhui ZHANG, Liming YUAN

**Affiliations:** 云南师范大学化学化工学院,云南 昆明 650500; School of Chemistry and Chemical Engineering, Yunnan Normal University, Kunming 650500, China

**Keywords:** 二维手性金属有机框架纳米片, 手性固定相, 气相色谱, 手性分离, two-dimensional chiral metal-organic-framework nanosheets, chiral stationary phase, gas chromatography (GC), chiral separation

## Abstract

二维金属有机框架材料(2D-MOFs)具有易于衍生化、孔隙率高、活性位点充足等特点,目前,鲜有将2D-MOFs应用于色谱分离的研究报道,在气相色谱手性分离分析领域的报道更为罕见。本文通过表面活性剂辅助溶剂热法制备了2D-MOFs纳米片,即Co-BDC-NH_2_,采用扫描电子显微镜、X射线粉末衍射对其进行表征。通过后修饰法分别将手性配体甘氨酰-L-天冬氨酸、甘氨酰-L-谷氨酸引入到Co-BDC-NH_2_中,得到两种具有手性的2D-MOFs纳米片材料,采用傅里叶红外光谱(FT-IR)、圆二色谱(CD)、热重分析(TGA)对其进行表征。将两种2D-MOFs纳米片材料用作气相色谱手性固定相,采用动态涂覆法分别将其涂覆到石英毛细管柱中,通过扫描电子显微镜分析,确定制备得到两根手性柱,并进行气相色谱分离测试。结果表明:两根色谱柱的理论塔板数各为3538和3108 N/m,柱效优良;两根柱子的麦氏常数依次为181与208,说明两种手性2D-MOFs纳米片是中等极性。两根色谱柱对位置异构体、外消旋化合物(尤其是氨基酸衍生物类)表现出较好的分离能力,其中,Co-BDC-NH_2_-甘氨酰-L-天冬氨酸与Co-BDC-NH_2_-甘氨酰-L-谷氨酸色谱柱分别对7种与8种外消旋化合物表现出手性识别能力。此外,Co-BDC-NH_2_-甘氨酰-L-天冬氨酸色谱柱对正构烷烃混合物、正构醇混合物及Grob试剂也表现出较好的分离效果。本研究在2D-MOFs纳米片上引入了手性功能团,验证其作为气相色谱固定相的可行性,也拓展了其在手性分离分析领域的应用。

## Abstract (en)

Two-dimensional metal-organic-framework (2D-MOF) materials have emerged as a new class of functional 2D material. Compared to bulk crystals, 2D-MOFs are easily derivatized, highly porous, and have sufficient active sites. While 2D-MOFs are of considerable research interest, they are also efficient candidates for multiple applications in a variety of fields owing to their numerous advantages. The ability to separate and analyze chiral compounds is greatly significant for progressing human society, and chromatographic separation is widely used in this regard owing to its high resolution and sensitivity. Few reports on the use of 2D-MOFs in chromatographic-separation applications currently exist, and those use gas chromatography to analyze and separate enantiomers are even rarer. Unsurprisingly, the development of novel stationary phases has become a popular topic in the chiral-chromatography field. In this study, 2D-MOF nanosheets (Co-BDC-NH_2_) were synthesized using a surfactant-assisted solvothermal method. The nanosheets were subsequently characterized by scanning electron microscopy and X-ray diffractometry. MOFs can be post-synthetically modified without affecting their frameworks, and such modifications can lead to the construction of chiral MOFs. Accordingly, Co-BDC-NH_2_ was post-synthetically modified with glycyl-L-aspartic acid and glycyl-L-glutamic acid as chiral ligands to afford two chiral 2D-MOF nanosheets, namely Co-BDC-NH_2_-glycyl-L-aspartic acid and Co-BDC-NH_2_-glycyl-L-glutamic acid. These chiral 2D-MOF nanosheets were characterized by Fourier-transform infrared spectroscopy, circular dichroism, and thermogravimetric analysis. The two 2D-MOF nanosheet materials were used as chiral stationary phases in gas chromatography by coating them onto prepared capillary columns using a dynamic coating method; this process leaves a homogeneous coating layer on the inner wall of the column. Scanning electron microscopy confirmed that the two chiral columns had been successfully prepared. The columns were finally tested through gas-chromatography-separation experiments. Theoretical plates can be used to evaluate column efficiency. The Co-BDC-NH_2_-glycyl-L-aspartic acid and Co-BDC-NH_2_-glycyl-L-glutamic acid columns were determined to have 3538 and 3108 N/m theoretical plates, respectively, which implies that these columns are highly efficient. The McReynolds constant can be used to determine the polarity of the stationary phase in a chromatographic column; the two capillary columns exhibited McReynolds constants of 181 and 208, consistent with materials of medium polarity. The two chromatographic columns exhibited good abilities to resolve positional isomers and racemates (especially amino-acid derivatives), with seven racemates identified using the Co-BDC-NH_2_-glycyl-L-aspartic acid column, and eight identified using the Co-BDC-NH_2_-glycyl-L-glutamic acid column. In addition, the former column also separated mixed *n*-alkanes, mixed *n*-alcohols, and Grob mixtures. In this study, we augmented 2D-MOF nanosheets with chiral functional groups and confirmed that they are effective stationary phases for use in gas-chromatography applications. The study also expands the applicability of 2D-MOF nanosheets to chiral separation.

手性(chirality)一词源于希腊语中的“*cheir*”,它类似于人手的关系,即两者互为镜像,且不相重合,如大自然中的贝壳,生命体中的蛋白质、多糖等都是手性的,手性涉及各种生命的生存与进化,在自然界中无所不在^[1]^。气相色谱法是一种应用广泛的手性拆分方法,该方法具有分析节奏快、分析效能高、应用范围广泛和分离与测定可同时进行等特点^[2]^。气相色谱法对挥发性较强的小分子具有较高的分辨率和灵敏度,基于手性固定相的气相色谱法已成为分离手性物质的优选方法^[3]^,固定相的性质决定了色谱柱能否满足对各种分析物的高选择性和高灵敏度的检测需求^[4]^,随着气相色谱法在手性分离分析领域的发展,开发新型的手性固定相材料变得尤为重要。

金属有机框架材料(MOFs)是一种新型的可调节化学成分与结构的多孔配位聚合物,与传统的多孔材料相比,其热稳定性、比表面积都有所提升,空隙结构也更加有序,故被广泛应用于气体吸附^[5]^、催化^[6]^与分离^[7]^等领域。2010年,严秀平等^[8]^制备了MIL-101涂覆的毛细管柱,首次将MOFs作为毛细管气相色谱固定相进行研究,在1.6 min内实现了对二甲苯、邻二甲苯、间二甲苯和乙苯的基线分离。手性金属有机框架材料(CMOFs)以其良好的结构精度、明确的手性功能、可调节的孔道尺寸、多样化手性识别位点等诸多独特优势,在手性分离分析领域得到了广泛关注^[9]^。CMOFs现已被大量用于色谱分离技术中^[10]^,目前,CMOFs的合成途径主要有直接合成法、合成后修饰法、手性模板诱导法3种^[11]^。直接合成法是指直接通过对映体纯配体或手性MOFs自发拆分合成手性MOFs;合成后修饰法是指在非手性的MOFs骨架中植入手性基团;手性模板诱导法则是通过选用恰当的非手性模板诱导构建手性MOFs。2011年,袁黎明等^[12]^首次将手性MOFs应用于气相色谱分离研究中,将具有三维手性孔道结构的手性MOFs [Cu(sala)]*_n_*作为固定相,对映选择性和拆分能力是该固定相最突出的优点。2021年,谢生明等^[13]^采用后修饰法使用手性聚苯胺对非手性MOFs表面进行改性,合成了手性核壳复合材料(MIL-101@c-PANI),将其用于高效液相色谱填充柱固定相,分离了醇、酮、酯、醛、有机酸和胺等12种手性化合物,该色谱柱对位置异构体的分离也表现出良好的选择性。2021年,谢生明等^[14]^合成了手性MOFs [Ni(S-mal)(bpy)]*_n_*,并将其作为高效液相色谱手性固定相。该固定相对氨氯地平、布洛芬、氟比洛芬、盐酸心得安、马来酸氯非那明和1-对氯苯-乙醇6种外消旋化合物具有良好的手性识别能力。

二维纳米材料主要包括氧化石墨烯(GO)^[15]^、二维共价有机框架材料(2D-COFs)^[16]^、二维金属-有机框架材料(2D-MOFs)^[17]^等。它们共同的特点就是具有较好的平面结构和高比表面积,且非常适合自组装和衍生化。GO本身就具有二维结构,已报道的COFs也以2D-COFs材料占大多数,而MOFs只有少部分是二维材料。2D-MOFs具有纳米级厚度、可横向延伸等特点^[18]^,还保留了MOFs材料的优良多孔结构。扩展的横向尺寸和纳米级厚度赋予了2D-MOFs较大的比表面积和无数可接近的表面活性位点^[19]^,使它们在气体分离^[20]^、催化^[21]^、传感^[22]^、药物输送^[23]^、染料去除^[24]^等领域有巨大的应用潜力。目前2D-MOFs已经被用于多种分离膜的研究^[20,25]^。2021年,古志远等^[26]^成功合成了3种不同形态的二维纳米材料,并探讨了其作为气相色谱固定相的进一步应用,由于扩散屏障的减少和在毛细管柱上得到了更均匀的涂层,3种材料表现出了良好的分离性能。然而,将2D-MOFs应用于气相色谱手性固定相的报道还很少。

本文先通过溶剂热法合成具有活性基团氨基(-NH_2_)的2D-MOFs,即Co-BDC-NH_2_,再将甘氨酰-L-天冬氨酸和甘氨酰-L-谷氨酸依次修饰到Co-BDC-NH_2_上,从而得到两种手性2D-MOFs纳米片材料:Co-BDC-NH_2_-甘氨酰-L-天冬氨酸和Co-BDC-NH_2_-甘氨酰-L-谷氨酸。采用动态涂覆法将两种手性2D-MOFs纳米片材料分别涂覆到石英毛细管柱中,使其在柱内壁形成一层固定相薄膜,通过气相色谱法考察两根色谱柱的分离能力。结果表明两根色谱柱对位置异构体和手性物质尤其是氨基酸衍生物具备较好的拆分能力,而且两根色谱柱表现出了良好的互补性。本研究说明了向2D-MOFs纳米片材料中引入手性功能团的可行性,也展现出了将2D-MOFs纳米片材料作为气相色谱手性固定相研究的巨大潜力。

## 1 实验部分

### 1.1 仪器与试剂

GC-2014气相色谱仪(岛津公司,日本); Hitachi Regulus8100扫描电子显微镜(日立公司,日本); X'Pert PRO MPD粉末衍射仪(帕纳科公司,荷兰); Nicolet Nexus 470红外光谱仪(Perkin Elmer公司,美国); Chirascan Plus圆二色谱仪(Applied Photophysics公司,英国); SDT-650热重分析仪(Taber公司,美国)。

三吡咯烷基溴化鏻六氟磷酸盐(PyBrOP)、2-氨基对苯二甲酸、*N*,*N*-二甲基甲酰胺(DMF)、4-二甲氨基吡啶(DMAP)、二氯甲烷以及测试过程中用到的化学试剂均购于上海阿拉丁生化科技股份有限公司;六水硝酸钴、乙醇、聚乙烯吡咯烷酮(PVP)均购于上海麦克林生化科技股份有限公司;甘氨酰-L-天冬氨酸购于日本TCI公司;甘氨酰-L-谷氨酸购于上海阿达马斯试剂公司。实验中所用到的*N*,*N*-二甲基甲酰胺的纯度为99.5%,乙醇的纯度为99.7%,其余试剂的纯度均为99.0%。

### 1.2 Co-BDC-NH_2_的合成

参考文献[28],称取0.0714 g 2-氨基对苯二甲酸溶于40 mL DMF-乙醇(1∶1, v/v)溶液中;称取0.250 g Co(NO_3_)_2_·6H_2_O溶于20 mL去离子水中。然后在恒定磁力搅拌下将两种溶液混合均匀得到稳定溶液,加入0.25 g PVP,搅拌均匀后移入100 mL聚四氟乙烯反应釜内衬中密封后放入烘箱,在80 ℃下反应60 h,冷却后用蒸馏水和无水乙醇进行洗涤除杂,干燥备用。

### 1.3 两种手性2D-MOFs纳米片的合成

称取0.26 g甘氨酰-L-天冬氨酸(甘氨酰-L-谷氨酸)溶于10 mL无水二氯甲烷中,再加入0.60 g PyBrOP,充分搅拌得到稳定溶液,移入洁净的25 mL两颈烧瓶中,在25 ℃下反应1.2 h后加入0.31 g DMAP及0.1 g Co-BDC-NH_2_,继续反应4天,最后用蒸馏水洗涤除杂,干燥得到Co-BDC-NH_2_-甘氨酰-L-天冬氨酸(Co-BDC-NH_2_-甘氨酰-L-谷氨酸),命名为CMOF1 (CMOF2)。

### 1.4 毛细管气相色谱柱的制备

取2根毛细管柱(15 m×250 μm),对其进行粗糙化处理:在N_2_推动下依次用1 mol/L NaOH溶液(2 h)、去离子水(1 h)、0.1 mol/L HCl溶液(0.5 h)、去离子水(2 h)冲洗毛细管柱内壁,最后用N_2_吹出多余液体。处理完的毛细管柱在130 ℃柱温下氮气吹扫3.5 h。

采用动态涂覆法将CMOF1、CMOF2材料分别涂进2根已粗糙化的毛细管柱中:取少量固定相材料用研钵磨细,将其分散在无水乙醇中静置约40 min,无明显沉降后,取中上层液体与配制好的4.0 mg/mL聚硅氧烷OV-1701无水乙醇溶液等体积混合,通过超声去除气泡,接着在气相色谱仪中借助载气(N_2_)将固定液缓慢压入毛细管柱,待固定液完全充满柱子后,适当降低载气流速,继续将柱子吹干,最后通过程序升温对该柱子进行老化,即从35 ℃开始,以1.5 ℃/min升到180 ℃,并在此温度下保持2 h。

### 1.5 实验条件

载气:高纯氮气;燃气:氢气,65 kPa;助燃气:空气,51 kPa:进样口温度:250 ℃;检测器温度:250 ℃;分流比:50∶1。

## 2 结果与讨论

### 2.1 固定相表征

对Co-BDC-NH_2_进行SEM表征,图1a高倍率扫描电镜图中显示了其纳米片形态,具有优良的平面结构及多孔结构;图1b低倍扫描电镜图可观察到其为相互堆叠的片状结构。

**图1:**
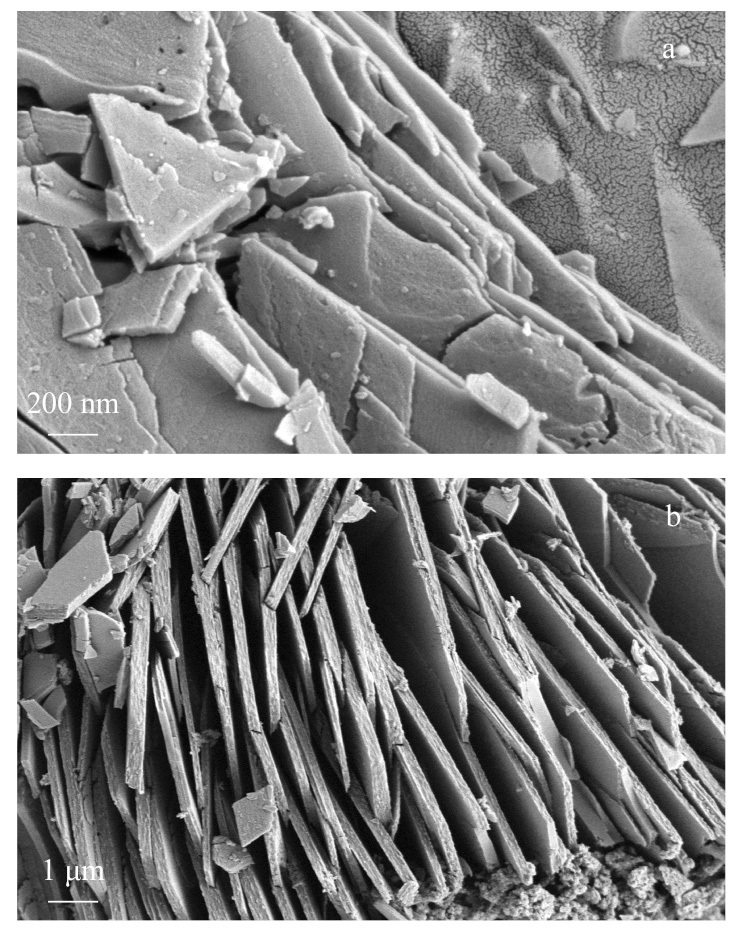
Co-BDC-NH_2_的扫描电镜图

对两种固定相进行XRD测试,观察到测试数据与晶体数据库模拟结果基本一致,修饰后的晶体结构也未发生变化。由图2可知,Co-BDC-NH_2_-甘氨酰-L-天冬氨酸在2*θ*=6.96°、9.35°、10.88°、17.93°与23.64°的位置出峰;Co-BDC-NH_2_-甘氨酰-L-谷氨酸在2*θ*=6.90°、9.30°、10.83°、17.96°与23.65°处出峰。

**图2:**
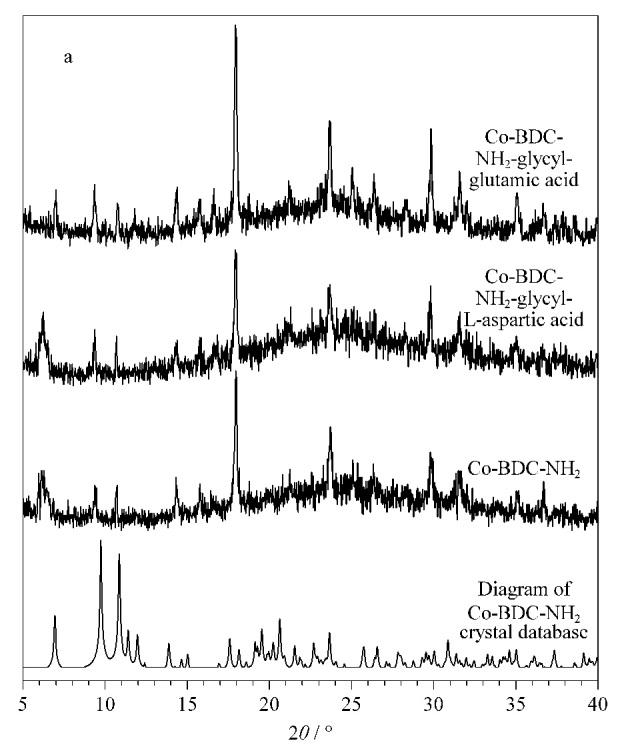
CMOF1和CMOF2的X射线衍射图谱

图3为两种材料的红外光谱对比图。BDC-NH_2_曲线在3595~3512 cm^-1^处出现-NH_2_吸收峰,而Co-BDC-NH_2_曲线在此区间的峰并不明显,这说明了BDC-NH_2_与Co^2+^反应时的去质子化,且Co-BDC-NH_2_曲线在1380 cm^-1^左右均出现芳香胺的伸缩峰与776 cm^-1^左右出现了Co-O键的吸收峰,说明已成功合成了Co-BDC-NH_2_。甘氨酰-L-天冬氨酸曲线在1686~1567 cm^-1^处有-COOH特征峰,Co-BDC-NH_2_-甘氨酰-L-天冬氨酸曲线在此区域的特征吸收峰得到增强,说明甘氨酰-L-天冬氨酸连接在-NH_2_上;甘氨酰-L-谷氨酸曲线在1709~1573 cm^-1^处有-COOH吸收峰,Co-BDC-NH_2_-甘氨酰-L-谷氨酸曲线在此区间特征吸收峰有所增强,说明甘氨酰-L-谷氨酸连接在-NH_2_上。

**图3:**
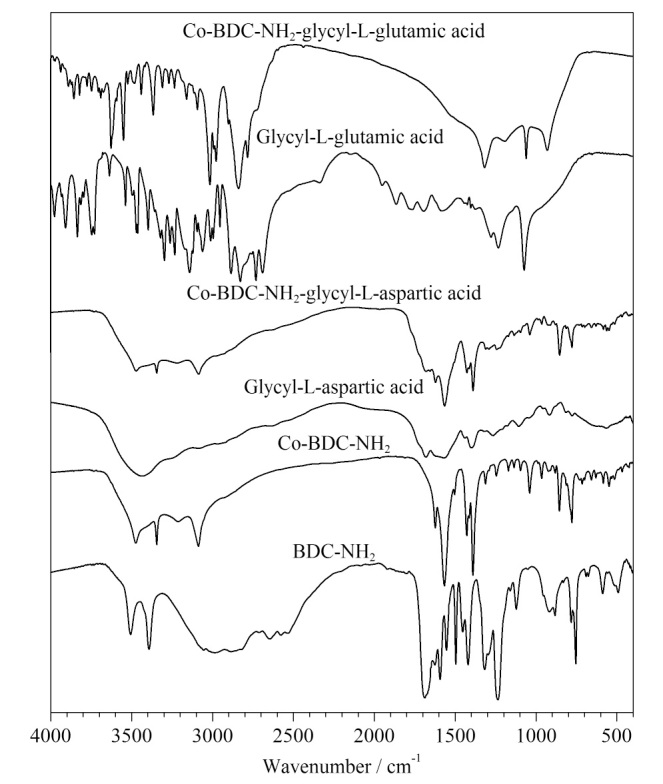
CMOF1和CMOF2的红外光谱图

为了确认两个CMOFs是否有手性,我们对其进行了圆二色谱测试,结果表明,两个固定相都产生了Cotton效应,说明它们具有手性(见图4)。

**图4:**
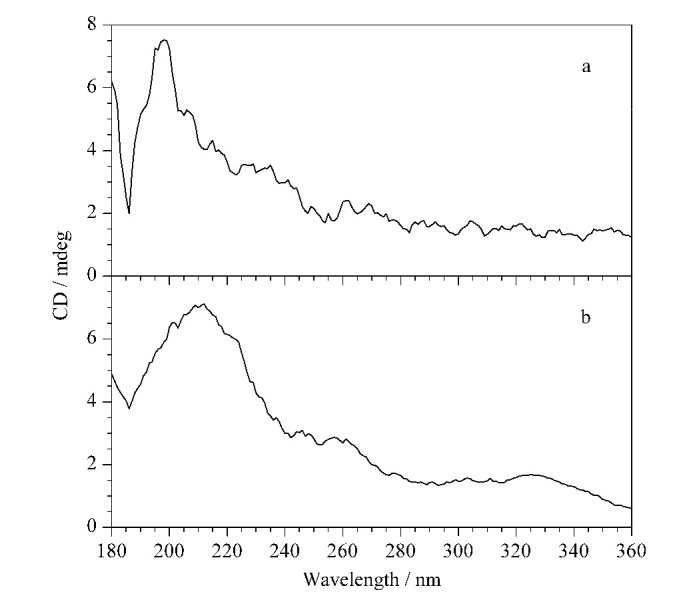
(a) CMOF1和(b) CMOF2的圆二色谱分析

对两个CMOFs进行热重分析来评估它们的热稳定性,结果如图5所示。由图5a可知,Co-BDC-NH_2_-甘氨酰-L-天冬氨酸在250 ℃前几乎没有损失,表明其热稳定性好;由图5b可看出,Co-BDC-NH_2_-甘氨酰-L-谷氨酸的稳定性不如Co-BDC-NH_2_-甘氨酰-L-天冬氨酸,从300 ℃开始其逐渐被分解,这可能是由于晶体中的结晶水挥发导致了晶体质量的流失。

**图5:**
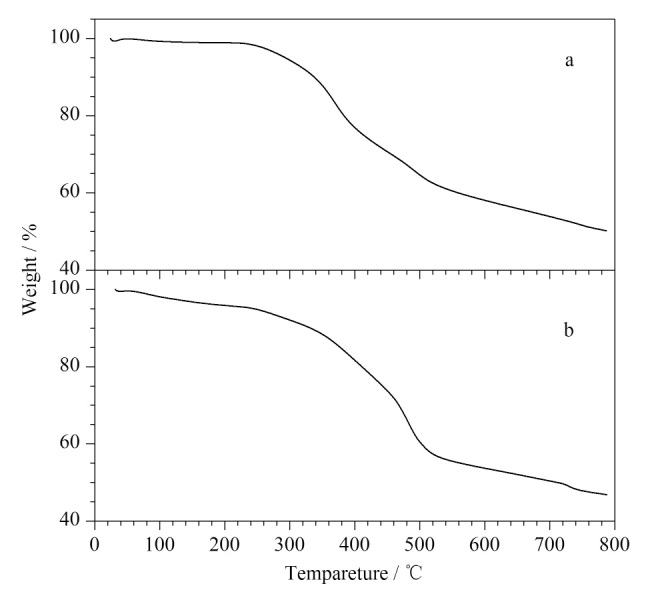
(a) CMOF1和(b) CMOF2的热重分析曲线

### 2.2 两根手性毛细管气相色谱柱的电镜表征

为观察固定相在毛细管柱中的涂覆情况,我们在两根色谱柱中分别截取长约1 cm的毛细管柱,对截面进行扫描电子显微镜测试,如图6所示,可以明显看到两种材料均成功涂覆在毛细管柱内壁,相较之下,CMOF1的涂覆情况更为理想。

**图6:**
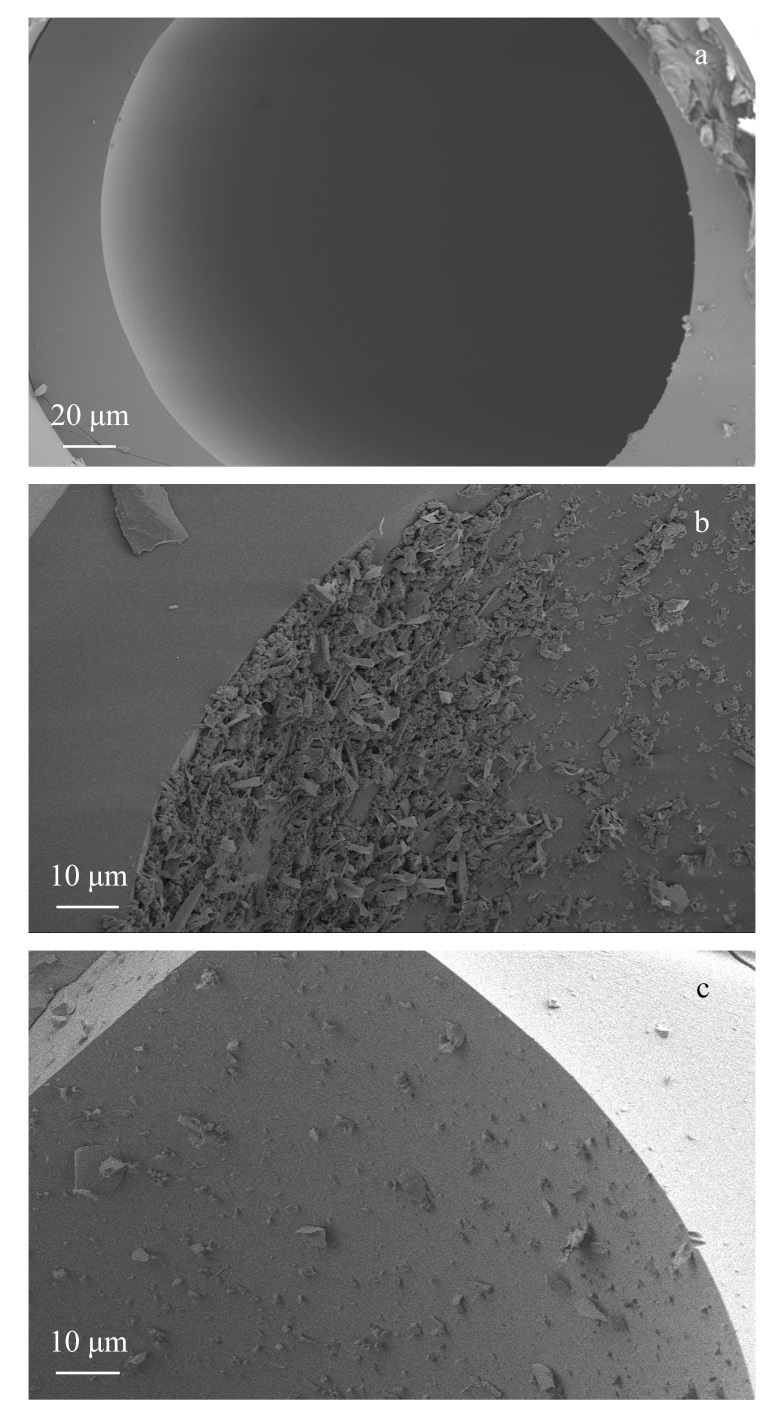
(a)空柱、(b) CMOF1色谱柱和(c) CMOF2色谱柱的扫描电镜图

### 2.3 两根手性毛细管气相色谱柱的柱效与极性评价

用正十二烷作测试物,在120 ℃下测试了两根色谱柱的理论塔板数,测定数据如表1,得到CMOF1与CMOF2柱的理论塔板数各为3538和3108 N/m,说明两根色谱柱的柱效优良。

**表1:** CMOF1、CMOF2色谱柱的性能

Column	Temperature/ ℃	Retention factor (*k*)	Linear velocity/ (cm/s)	Column efficiency/ (N/m)
CMOF1	120	1.43	14.5	3538
CMOF2	120	1.34	13.3	3108

实验选择1-丁醇等5种物质在120 ℃下进行测试,得到Co-BDC-NH_2_-甘氨酰-L-天冬氨酸柱与Co-BDC-NH_2_-甘氨酰-L-谷氨酸柱的麦氏常数依次为181与208(见表2),说明这两种CMOFs为中等极性固定相。

**表2:** CMOF1、CMOF2色谱柱的麦氏常数

Column	Benzene	2-Pentanone	1-Butanol	1-Nitropropane	Pyridine	Average polarity
CMOF1	102	157	195	238	211	181
CMOF2	168	179	225	213	254	208

### 2.4 正构烷烃在CMOF1色谱柱上的分离

表3为CMOF1色谱柱对正构烷烃混合物(*n*-C_6_~*n*-C_10_)的分离数据,表明分离效果良好。升温程序:52 ℃保持1 min, 50 ℃/min升到210 ℃; N_2_流速:18.7 cm/s。

**表3:** 正构烷烃在CMOF1色谱柱上的分离数据

*n*-Alkane	Separation factor (*α*)^*^	Resolution (*R*_s_)^*^
*n*-Hexane	-	-
*n*-Heptane	1.73	1.52
*n*-Octane	1.89	2.46
*n*-Nonane	1.97	3.44
*n*-Decane	1.72	4.14

* With the previous adjacent peak.

### 2.5 正构醇在CMOF1色谱柱上的分离

表4为CMOF1色谱柱对正构醇混合物(*n*-C_1_~*n*-C_9_)的分离数据,表明分离效果良好。升温程序:50 ℃,保持1 min, 55 ℃/min升到200 ℃; N_2_流速:18.7cm/s。

**表4:** 正构醇在CMOF1色谱柱上的分离数据

*n*-Alcohol	*α*	*R*_s_	
Methanol	-		-
Ethanol	4.28		1.03
1-Propanol	2.36		1.28
1-Butanol	2.34		2.04
1-Pentanol	1.61		1.90
1-Hexanol	1.36		1.90
1-Heptanol	1.24		1.98
1-Octanol	1.29		4.37
1-Nonanol	1.10		1.64

### 2.6 Grob试剂在CMOF1色谱柱上的分离

Grob试剂常用作评价气相色谱柱整体分离性能,表5为CMOF1色谱柱对Grob试剂的分离数据,表明CMOF1色谱柱能较好分离11种Grob试剂。升温程序:45 ℃,保持1 min, 50 ℃/min升到200 ℃; N_2_流速:17.5 cm/s。

**表5:** Grob试剂在CMOF1色谱柱上的分离数据

Grob	*α*	*R*_s_	Grob	*α*	*R*_s_	Grob	*α*	*R*_s_
2,3-Butanediol	-	-	2,6-Xylidine	1.21	1.65	Dicyclohexylamine	1.11	1.78
*n*-Decane	3.66	2.50	2,6-Xylenol	1.10	0.76	Methyl undecanoate	1.09	1.57
*n*-Undecane	1.98	3.03	1-Nonanal	1.08	0.57	Methyl laurate	1.10	1.33
1-Octanol	1.95	5.79	Methyl caprate	1.06	0.73			

### 2.7 位置异构体在色谱柱上的分离

选用几种位置异构体分别在CMOF1与CMOF2色谱柱上进行测试,实验数据见表6与表7,拆分图见图7与图8, CMOF1色谱柱能分开3种异构体,CMOF2色谱柱能分开4种异构体,且二者不仅展现出各自的选择性,还起到了相互补充的作用。

**表6:** 位置异构体在CMOF1色谱柱上的分离数据

Positional isomers	*T*/℃	*k*_1_	*k*_2_	*α*_1_	*α*_2_	*R*_s1_	*R*_s2_	*v*/(cm/s)
*α*,*β*-Ionone	150	1.26	1.32	1.04	-	0.59	-	13.9
*cis*-/*trans*-Citral	125	1.13	1.19	1.05	-	0.62	-	15.6
*o*,*m*,*p*-Nitrobromobenzene	125	0.69	0.81	1.17	1.03	2.31	0.67	12.5

*k*_1_: retention factor of the first peak; *k*_2_: retention factor of the second peak; *α*_1_: separation factor of the first peak and the second peak; *α*_2_: separation factor of the second peak and the third peak; *v*: linear velocity of carrier gas N_2_.

**表7:** 位置异构体在CMOF2色谱柱上的分离数据

Positional isomers	*T*/℃	*k*_1_	*k*_2_	*α*_1_	*α*_2_	*R*_s1_	*R*_s2_	*v*/(cm/s)
*α*,*β*-Ionone	128	1.23	1.33	1.08	-	0.89	-	12.6
*o*,*m*,*p*-Dinitrobenzene	135	0.73	0.77	1.06	1.10	0.75	0.97	12.2
*o*,*m*,*p*-Chlorophenol	110	0.86	0.91	1.06	1.53	0.63	2.82	15.1
*o*,*m*,*p*-Nitrobromobenzene	110	0.93	1.12	1.20	1.04	3.52	0.66	14.2

**图7:**
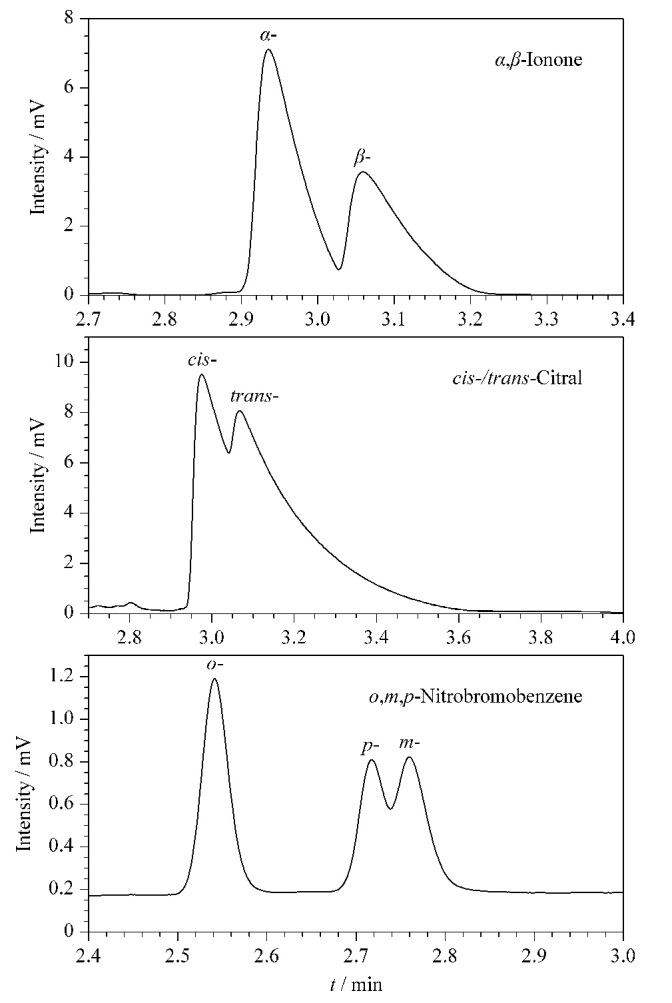
位置异构体在CMOF1柱上的色谱图

**图8:**
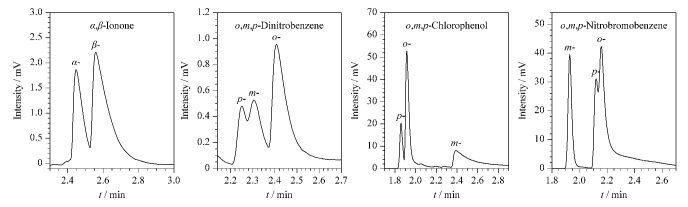
位置异构体在CMOF2柱上的色谱图

### 2.8 外消旋体化合物在色谱柱上的分离

CMOF1、CMOF2色谱柱对外消旋化合物的分离主要利用对映体分子与固定相的氢键作用强度差异来实现手性拆分。此外,偶极-偶极作用、空间立体作用和范德华力等对手性分离也有一定的影响。通过测试部分外消旋体化合物在两根柱子上的拆分情况,我们考察到CMOF1色谱柱对7种手性物质有识别能力,尤其是对丙氨酸的拆分效果好,*R*_s_值能达到4.93,实验数据见表8,拆分图见图9; CMOF2色谱柱能拆分8种手性物质,其对醇类的拆分优于前者,实验数据见表9,拆分图见图10。另外,CMOF1、CMOF2色谱柱对氨基酸类物质的识别效果存在一定的相似性与互补性。

**表8:** 外消旋体在CMOF1色谱柱上的分离

Racemate	*T*/℃	*k*_1_	*α*	*R*_s_	*v*/(cm/s)
DL-Alanine^a^	150	0.72	2.59	4.93	13.0
2-Methylpentanoic acid	178	0.91	1.06	0.79	14.0
2-Methylvaleraldehyde	178	0.90	1.24	1.84	13.1
Menthol	155	1.01	1.09	1.29	15.0
DL-Lysine^a^	162	1.25	1.06	0.99	13.5
DL-Methionine^a^	172	1.57	1.16	1.15	15.6
Butyl glycidyl ether	133	0.99	1.14	1.67	14.2

a: trifluoroacetyl isopropyl ester derivative.

**图9:**
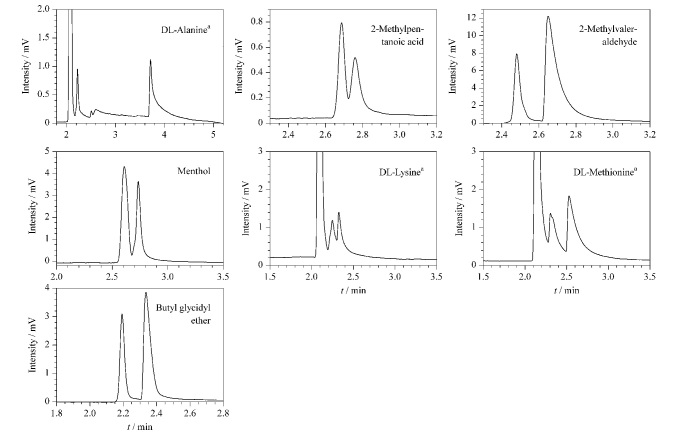
外消旋体在CMOF1柱上的色谱图

**表9:** 外消旋体在CMOF2色谱柱上的分离

Racemate	*T*/℃	*k*_1_	*α*	*R*_s_	*v*/(cm/s)
Butyl glycidyl ether	110	0.70	2.17	3.92	14.4
1,2-Epoxybutane	100	1.02	1.04	0.58	14.7
Styrene oxide	100	1.29	1.08	0.74	13.9
Methyl phenyl sulfoxide	127	0.76	1.23	1.36	14.1
Menthol	129	0.63	1.73	4.74	14.5
Limonene	150	1.06	1.06	0.71	14.9
DL-Methionine^a^	185	1.06	1.13	0.99	13.5
DL-Isoleucine^a^	145	0.96	1.11	0.68	15.5

**图10:**
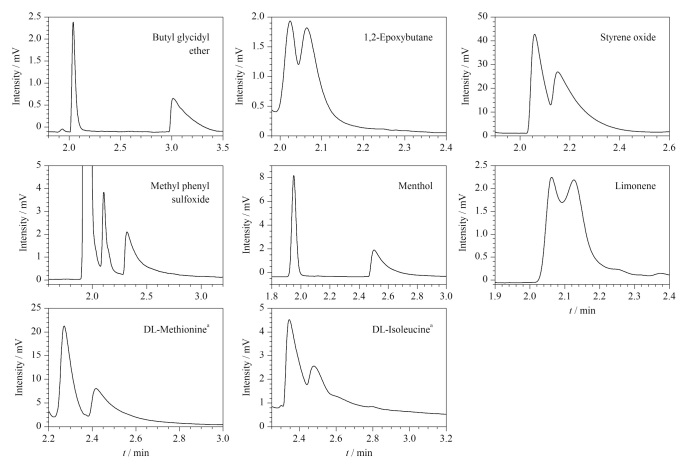
外消旋体在CMOF2柱上的色谱图

### 2.9 两根手性毛细管柱的稳定性与重复性

分别选取0.02、0.04、0.06、0.08、0.10 μL 2-甲基戊醛和0.01、0.05、0.10、0.15、0.20 μL氧化苯乙烯,其他条件保持一致,重复进样5次,测试CMOF1与CMOF2色谱柱在不同进样量时的分离效果。经分析,2-甲基戊醛、氧化苯乙烯保留时间的相对标准偏差分别为1.53%和1.32%。然后将2-甲基戊醛和氧化苯乙烯分别在CMOF1与CMOF2色谱柱上初次分离与两柱经过400次左右的快速升温降温过程后的分离情况进行比较,2-甲基戊醛、氧化苯乙烯保留时间的相对标准偏差分别为0.816%和0.690%。结果表明,两根毛细管柱都有良好的稳定性与重复性。

## 3 结论

本文通过后修饰法得到了两种手性二维金属有机框架纳米片,即Co-BDC-NH_2_-甘氨酰-L-天冬氨酸与Co-BDC-NH_2_-甘氨酰-L-谷氨酸,将它们用作气相色谱手性固定相,制得CMOF1与CMOF2色谱柱,两柱均表现出较好的分离能力,具体表现如下:首先,两根色谱柱对位置异构体和手性物质外消旋体展现出不同的拆分效果,具有一定的互补性;拆分能力优异,可扩展手性二维金属有机框架材料对位置异构体及外消旋体的拆分范围。其次,CMOF1色谱柱对正构烷烃、正构醇混合物及Grob试剂都表现出较好的分离性能。最后,从所拆分的外消旋体的数目和种类上比较,CMOF2色谱柱的表现更突出。综上,二维金属有机框架作为气相色谱固定相具有较大的研究价值,将二维金属有机框架材料与手性功能团结合应用于手性分离也有较好的发展前景。
